# Postlingual Hearing Loss as a Mitochondrial 3243A>G Mutation Phenotype

**DOI:** 10.1371/journal.pone.0044054

**Published:** 2012-10-25

**Authors:** Katarzyna Iwanicka-Pronicka, Agnieszka Pollak, Agata Skórka, Urszula Lechowicz, Magdalena Pajdowska, Mariusz Furmanek, Maciej Rzeski, Lech Korniszewski, Henryk Skarżyński, Rafał Płoski

**Affiliations:** 1 Institute of Physiology and Pathology of Hearing, Warsaw, Poland; 2 World Hearing Center, Kajetany, Poland; 3 Department of Medical Genetics, Medical University of Warsaw, Poland; 4 Department of Paediatrics, Medical University of Warsaw, Poland; 5 Department of Biochemistry and Experimental Medicine, The Children's Memorial Health Institute, Warsaw, Poland; Oslo University Hospital, Norway

## Abstract

**Background:**

The prevalence of isolated hearing loss (HL) associated with the m.3243A>G mutation is unknown. The aim of this study was to assess the frequency and heteroplasmy level of the m.3243A>G mutation in a large group of Polish patients with postlingual bilateral sensorineural HL of unidentified cause.

**Methodology/Principal Findings:**

A molecular search was undertaken in the archival blood DNA of 1482 unrelated patients with isolated HL that had begun at ages between 5 and 40 years. Maternal relatives of the probands were subsequently investigated and all carriers underwent audiological tests. The m.3243A>G mutation was found in 16 of 1482 probands (an incidence of 1.08%) and 18 family members. Of these 34 individuals, hearing impairment was detected in 29 patients and the mean onset of HL was at 26 years. Some 42% of the identified m.3243A>G carriers did not develop multisystem symptomatology over the following 10 years. Mean heteroplasmy level of m.3243A>G was lowest in blood at a level of 14% and highest in urine at 58%. These values were independent of the manifested clinical severity of the disease.

**Conclusions:**

A single m.3243A>G carrier can usually be found among each 100 individuals who have postlingual hearing loss of unknown cause. Urine samples are best for detecting the m.3243A>G mutation and diagnosing mitochondrially inherited hearing loss.

## Introduction

Postlingual hearing loss (HL) occurs in 85–95% of patients with multisystem syndromes associated with an m.3243A>G mutation in the tRNA^leu^ (UUR) gene [1]–[4]. In the MELAS phenotype (mitochondrial encephalomyopathy lactic acidosis stroke-like episodes) [5], [6], HL is the fourth most frequent of 17 symptoms [7]; in the MIDD phenotype (mitochondrially inherited diabetes-deafness syndrome), hearing impairment is usually the second most-frequent symptom [8], [9]. The origin of the mitochondrial sensorineural defect may be cochlear [3], [10] or retro-cochlear if there is central neurological involvement [11]. Major findings in the cochleas of subjects with the m.3243A>G mutation are degeneration and decreased cochlear neurons [12], fewer outer hair cells and endolymphatic sac cells [13], and atrophy of *stria vascularis* [12]–[14].

Although the prevalence of the most frequent m.3243A>G tRNA leucine (UUR) transition (MTTL1) in the general population is known [13], [15]–[17], and the occurrence of mitochondrial disease associated with various mtDNA mutations is estimated as more than 1 in 5,000 [18], knowledge of the frequency of m.3243A>G mutation associated with isolated hearing impairment is limited [19]–[23] since usually, only m.1555A>G mitochondrial 12S rRNA mutation, and a few other specific mtDNA mutations associated with aminoglycoside susceptibility, are tested in patients with nonsyndromic HL [24]–[26].

## Methods

### Objectives

The aim of the study was to assess the frequency and heteroplasmy level of the m.3243A>G mutation in a large group of Polish patients with postlingual bilateral sensorineural HL of unidentified cause.

### Patients

The study group was recruited from a cohort of 7000 individuals who consulted the Genetics Department of the Institute of Physiology and Pathology of Hearing (the Polish national referral centre) between the years 2000 and 2010. Only cases with bilateral HL, starting at an age between 5 and 40 years, and with available archived DNA sample, were included in the study. Patients were excluded if they had an established cause of hearing impairment, had syndromic HL of known genetic origin, or had two mutations in the connexin genes *GJB2* and *GJB6* (homozygosity or compound heterozygosity status) [27], [28]. In total, 1482 archived blood DNA samples were made available for the m.3243A>G mutation search.

The data retrieved from medical reports of the recruited patients included: (1) the age of the patient, (2) gender, (3) age of HL onset (defined as the age at which the patient or his/her parents first became aware of hearing impairment), (4) the result of the first audiological examination based on pure tone audiometry (PTA), (5) the degree of HL (defined as the arithmetic mean of the frequencies 0.5, 1, 2, and 4 kHz for the better-hearing ear), (6) whether the HL was progressive or stable (determined by comparing the mean hearing threshold of each patient's initial and last audiogram), (7) the presence of additional symptoms of dysfunction of the hearing pathway like tinnitus and/or vertigo, (8) other health problems of the patient, and (9) the family history of hearing impairment, the basic health status of maternal relatives with hearing problems, and sudden deaths in the family. Characteristics of the study group are shown in Table 1.

**Table 1:** Characteristics of the study group: all examined patients with hearing loss (HL), and the 34 patients identified as having the m.3243A>G mutation.

	Studied patients	m.3243A>G mutation carriers
Number	1482	34*
Males	649 (44%)	13 (38%)
Females	833 (56%)	21 (62%)
Mean age at the study (years)	5–62, mean 27.0	6–61, mean 31.0
Mean age of onset of HL (years)	5–40, mean 15; SD = 10.4	8–63, mean 26^1^ SD = 13.7; N = 29***
Profound degree of hearing loss**	3.8%	3.4%
Severe degree of hearing loss**	6.6%	13.8%
Moderate degree of hearing loss**	43.6%	51.8%
Mild degree of hearing loss**	46%	31%
Normal hearing	0	5
Other dysfunction of the inner ear (tinnitus/vertigo)	399/103; (27%/7%)	12/5; (35%/15%)
Progression of hearing loss during observation	ND	14
Positive family history of hearing loss	528 (35.6%)	33 (97.0%)^2^
Development of additional multi-organ 3243A>G pathology	ND	17
Number of 35delG *GJB2* heterozygotes	80 (5.5%)	1/16 (6.3%)

* 16 probands and 18 relatives.

** Arithmetic mean of 0.5, 1, 2 and 4 kHz for the better-hearing ear.

*** N = number of patients; 29 of 34 had HL; 5 subjects (aged 6–20) had normal hearing.

1 *p*<0.000001 vs patients without m.3243A>G (*t*-test).

2 *p*<0.00001.

ND = no data.

Clinical and audiological status of the carriers of the m.3243A>G mutation were re-assessed, and they underwent genetic counselling. Maternal relatives of the m.3243A>G probands were also invited for audiological and genetic consultation. Severity of mitochondrial disease was assessed according to the Newcastle Mitochondrial Diseases Adult Scale (NMDAS) [29].

### Clinical and laboratory investigations

Hearing levels were determined by pure tone audiometry. Evaluation of the degree of hearing loss was based on ANSI (American National Standards Institute) and ISO (International Standards Organization) standards. On these scales, mild hearing loss is that up to 40 dB HL, moderate is 41–70 dB HL, severe is 71–90 dB HL, and profound is more than 90 dB HL. Additional hearing tests – otoacoustic emissions, speech audiometry, tympanometry with stapedial reflexes, and brainstem evoked auditory potentials (BEAP) – were conducted to identify the character of the hearing loss (sensorineural hearing loss SNHL of cochlear origin, retro-cochlear, or conductive).

Cerebral alterations and brain lactates were assessed by magnetic resonance imaging with magnetic resonance spectroscopy (Siemens 3T). Organic acids excreted in urine were analysed using gas chromatography-mass spectroscopy (GC-MS).

Additional samples for the heteroplasmy study (urine sediment, nails, hair follicles, and buccal mucosa smear) were collected from individuals with confirmed or suspected m.3243A>G mutation and who gave signed consent.

A search for the m.3243A>G mutation was performed using TaqMan Assay on Demand from Applied Biosystems, Foster City, CA, according to the manufacturer's instructions. All positive samples were directly sequenced to confirm the presence of the mutation using primers M_F and M_R without 5′JOE modification (Table 2). The heteroplasmy level was assessed by PCR-RFLP using the primers and ApaI (Fermentas, Lithuania) enzyme (the m.3243A>G mutation creates a restriction site for this enzyme). Digested products were analyzed on an ABI 3130 capillary sequencer. Finally, by analysing the area under the peak the mutated/non-mutated DNA ratio was assessed.

**Table 2:** The sequences of the primers M_F and M_R.

Primer	Sequence	modification
M_F	5′CCTCCCTGTACGAAAGGACA3′	none
M_R	5′AGGAGTAGGAGGTTGGCCATGG 3′	5′ JOE

### Ethics statement

The bioethical commission of the Institute of Physiology and Pathology of Hearing approved the study and all subjects or their guardians gave signed consent.

### Statistical analysis

Comparisons of mean onset of HL among m.3243A>G carriers versus other patients were done using a student *t-*test for independent samples. Comparison of heteroplasmy levels in different tissues was performed by a student *t*-test for dependent samples. Correlation between total disease score and heteroplasmy levels was performed by linear regression. All analyses were done using the Statistica software package.

## Results

### Prevalence of m.3243A>G

Our study of 1482 archival DNA samples of HL patients revealed m.3243A>G mutation in 16 cases (1.08%) indicating that on average the m.3243A>G mutation was found in one of 92 examined patients with isolated HL that had begun between the ages of 5 and 40 years.

In comparison with the whole studied HL group, patients with the m.3243A>G mutation had similar age, gender, degree of HL, and frequency of symptoms or other dysfunctions of the inner ear. The major features of the subgroup positive for m.3243A>G were maternal inheritance in all families (except one) and relatively later onset of HL in the m.3243A>G subgroup (Table 1).

The total of 34 identified individuals carrying the m.3243A>G mutation included 16 probands and 18 of their relatives detected at the second step by molecular investigation. Presence of the m.3243A>G mutation was not previously tested nor suspected in any of these cases.

The study group consisted of 29 patients with m.3243A>G HL (16 probands and 13 relatives). Five m.3243A>G relatives (children and young females) without hearing problems, but testing positively for the m.3243A>G mutation, were analysed separately.

Over the course of 10 years of observations, 17 of 29 HL patients developed multi-system mitochondriopathy while 12 remained oligosymptomatic. Clinical characteristics of the studied patients are shown in Table 3.

**Table 3:** Characteristics of the patients carrying the m.3243A>G mutation patients 1–17 with multi-organ presentation (subgroup I), patients 18–29 with isolated hearing loss (subgroup II), and asymptomatic carriers 30–34 (subgroup III).

Patient number	Gen der	Age (yrs)	Age of onset of HL (yrs)	HL severity	HL progression	Disease severity (NMDAS scale)	Organic acids profile in urine (GC-MS)	Increased lactates in brain (MRS)	Brain MRI	Symptoms and onset	Remarks
1	F	20	19	mild	Yes	7	NP	NP	NP	RP (20), SS	Aminogly*
**2**	**F**	**43**	**32**	**mild**	**No**	**15**	**MGCA**	**2**	**IMGP**	**M (40), RP (43)**	**Episode of blindness lasting 2 days**
**3**	**M**	**12**	**8**	**mild**	**No**	**55**	**NP**	**NP (died)**	**NP (died)**	**H (8), M (8), N (8), S (11)**	**Died at age 12**
4	F	38	35	moderate	Yes	19	MGCA LA	2	MCA IMGP	H (35), N (37), SS	Pancreatitis*
**5**	**F**	**41**	**10**	**moderate**	**ND**	**20 (part 2 only)**	**NP**	**NP**	**NP**	**DM (15), H (40)**	
6	M	13	9	moderate	No	81	NP	NP	NP	H (9), M (9), N (9), S (9), RP (10)	
**7**	**M**	**29**	**24**	**moderate**	**Yes**	**26**	**NA**	**NP (pacemaker)**	**NP**	**C (24), M (24), SS**	**Stress** *
**8**	**M**	**27**	**20**	**moderate**	**ND**	**33 (part 2 only)**	**NP**	**NP (died)**	**NP**	**M (21), N (21), S (27), SS**	**Died at age 27**
**9**	**M**	**42**	**32**	**moderate**	**No**	**33**	**NA**	**1**	**MCA**	**DM (38), K (40), RP (42), I**	**Impaired spermatogenesis at biopsy, end-stage renal failure**
**10**	**M**	**52**	**40**	**moderate**	**No**	**77**	**MGCA**	**3**	**MCA Focal lesion after S in occ-temp-parietal area. IMGP**	**M (40), N (40), RP (45), S (45), C (45), DM (46), H (50), I, SS**	**Impaired spermatogenesis at biopsy**
**11**	**F**	**16**	**8**	**severe**	**Yes**	**38**	**NA**	**2**	**Diffuse bilateral lesions after S in frontal, occ-temp-parietal areas, insular cortex. IMGP MCA**	**N (8), S (8)**	**Stress** *
12	F	47	37	severe	Yes	14	MGCA	2	MCA IMGP	C (40), M (45), I	Aminogly*
13	F	72	47	severe	No	74	NP	NP	NP	H (47), M (47), N (47), C (50), DM (50)	
14	M	37	19	severe	Yes	4	NA	1	MCA IMGP	SS	Noise*
**15**	**F**	**28**	**19**	**profound**	**Yes**	**31**	**LA**	**NP (CI)**	**NP**	**N (25), M (25)**	**Aminogly** *
**16**	**F**	**38**	**22**	**profound**	**Yes**	**33**	**NA**	**2**	**MCA IMGP**	**H (30), DM (35), K (36), M (38), SS**	**End-stage renal failure. Stress** *
**17**	**F**	**51**	**17**	**profound**	**Yes**	**99**	**NP**	**NP (CI)**	**NP**	**H (20), C (40), DM (40), N (40), RP (40), S (40), M (49), I, SS**	**Aminogly** *
18	F	18	17	mild	No	6	NP	1	NA	-	
**19**	**F**	**24**	**10**	**mild**	**No**	**1**	**NP**	**NP**	**NP**	**-**	
20	F	24	23	mild	No	1	NA	1	NA	-	
21	F	35	34	mild	Yes	4	NA	1	IMGP	-	Stress*
22	F	49	48	mild	Yes	12	MGCA	0	IMGP	-	Stress*
**23**	**M**	**29**	**9**	**mild**	**No**	**1**	**NA**	**0**	**NA**	**-**	
24	F	78	63	moderate	No	34	NA	1	Focal spread vascuar lesions of white matter (non-specific).	-	NMDAS score probably false positive, connected with advanced age
**25**	**M**	**32**	**29**	**moderate**	**Yes**	**8**	**NA**	**1**		**-**	**Noise** *
26	M	33	33	moderate	Yes	11	NP	NP	NP	-	Stress*
**27**	**M**	**51**	**20**	**moderate**	**ND**	**NP**	**NP**	**NP**	**NP**	**-**	
28	F	49	42	severe	No	25 (13 of part I - QoL)	NA	1	MCA	-	
**29**	**F**	**61**	**25**	**profound**	**Yes**	**7**	**NA**	**NP (CI)**	**NP**	**-**	**Pregnancy** *
30	F	5	No HL	-	No HL	11	NA	NP	NP	-	Hair heteroplasmy 20.4%
31	F	10	No HL	-	No HL	24 (15 of part I - QoL)	NA	NP	NP	-	Buccal heteroplasmy 100%
32	F	10	No HL	-	No HL	0	MGCA	0	NA	-	
33	F	19	No HL	-	No HL	5	LA	1	IMGP	-	
34	M	8	No HL	-	No HL	0	NA	1	NA	-	Hair heteroplasmy 63.7%

Probands are shown in bold.

GC-MS = gas chromatography–mass spectroscopy; NMDAS = Newcastle Mitochondrial Disease Adult Scale; MGCA = 3-methylglutaconic aciduria; LA = lactic aciduria; HL = hearing loss; NP = not performed; NA = normal value; QoL = quality of life; CI = cochlear implant user; IMGP = increased mineralisation of globus pallidus; MCA = minimal cerebellar atrophy; MRS = magnetic resonance spectroscopy; MRS score: 0 = negative LA, 1 = uncertain LA, 2 = positive LA, 3 = strong LA.

C = cardiomyopathy; DM = diabetes mellitus; H = migraine; I = infertility; K = renal insufficiency; M = myopathy; N = peripheral neuropathy; RP = pigmentary degeneration of retina; S = stroke-like episodes; SS = short stature.

No HL = normal hearing.

* Stress = hearing deterioration following stressful event; Aminogly = hearing deterioration following aminoglycoside administration; Noise = hearing deterioration following noise exposure; Pancreatitis = hearing deterioration following acute pancreatitis; Pregnancy = hearing deterioration after pregnancy.

According to the clinical status, the m.3243A>G carriers were divided into three subgroups:

- Multi-symptomatic subgroup (17 patients)
- Isolated hearing loss subgroup (12 patients)
- Asymptomatic subgroup (5 individuals).

### Audiological assessment

**Figure 1:**
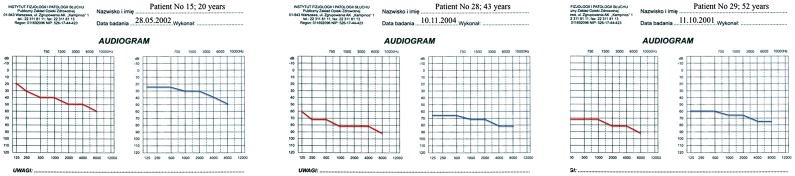
Audiograms of patients 15, 28, and 29 when first diagnosed with isolated hearing impairment.

Bilateral sensorineural hearing loss was the first presentation of the disease in all m.3243A>G patients (Figure 1). The mean age of onset of hearing impairment was 26 years: 18 years for men and 28 years for women (Table 4). HL started earlier in the multi-symptomatic subgroup (21 years) than in the isolated HL subgroup (32 years).

**Table 4:** Heteroplasmy levels (% of total tissue DNA) of m.3243A>G mutation in mtDNA samples isolated from: urinary sediment, hair follicles, buccal mucosa, nails and blood (put in order of heteroplasmy intensity).

No of patients in group; mean age	Females/Males	Gender-age of onset (mean age of onset in group)	No of studied samples of urinary sediment; Mean %; (Range %)	No of studied samples of hair follicles; Mean %; (Range %)	No of studied samples of buccal mucosa smear; Mean %; (Range %)	No of studied samples of nails; Mean %; (Range %)	No of studied samples of blood; Mean %; (Range %)
I group (17) 36 yrs	10/7	F–24 yrs; M–16 yrs (21 yrs)	**13**; 67%; (31.4%–96.5%)	**13**; 21.2%; (0%–77.2%)	**11**; 26.1%; (5.6%–60.2%)	**12**; 26.4%; (6.7%–65.2%)	**14**; 14,4%; (5,4%–27,8%)
II group (12) 40 yrs	8/4	F–37 yrs; M–23 yrs (32 yrs)	**11**; 51.5%; (7.6%–85.8%)	**8**; 25.8%; (1.6%–63.7%)	**10**; 21.0%; (1.2%–33.2%)	**9**; 14.2%; (1.8%–24.6%)	**12**; 9,3%; (0%–32,1%)
III group (5) 8 yrs	4/1	NA	**3**; 39.5%; (16.2%–81.3%)	**4**; 44.8%; (20.4%–66.5%)	**5**; 35.9%; (3.8%–100%)	**4**; 34.1%; (16.7%–49.6%)	**5**; 21,1%; (12,8%–32,5%)
Total 34 patients 33 yrs	22/12	F–28 yrs; M–18 yrs (26 yrs)	**27**; 57.6%; (7.6%–96.5%)	**26**; 26.5% (0%–77.2%)	**26**; 26.0%; (1.2%–100%)	**25**; 23.2%; (1.8%–65.2%)	**31**; 13,5%; (0%–32,5%)

F = female, M = male, yrs = years, NA = not applicable.

The subgroups of patients: I – multi-organ presentation, II - isolated hearing loss and III - asymptomatic are presented separately.

Progression of the HL was observed in 14 out of 29 (48%) patients: in 10 women and 4 men (Table 3). The proportion of patients with a progressive course of HL was similar in both multi-symptomatic and isolated HL subgroups (Table 5).

**Table 5:** Comparison of clinical status among all groups of patients (I, II and III) carrying m.3423A>G mutation: I = Multi-symptomatic subgroup, II = Isolated HL subgroup, III = Asymptomatic subgroup.

No of patients in groups	Mild degree of HL	Moderate degree of HL	Severe degree of HL	Profound degree of HL	Progression of HL	Mean value of NMDAS score (parts 1–3)	Normal value/Examined (GC-MS)	LA/MGCA (GC-MS)	0 = no brain LA (MRS)	1 = uncertain brain LA (MRS)	2 = positive brain LA (MRS)	3 = strong brain LA (MRS)	Brain MRI alterations/Examined	Heteroplasmy level in urine (%)	Heteroplasmy level in blood (%)
I (17)	3	7	4	3	53% 9/17	39*	5/10	2/4	0	2	5	1	8/8	67	14
II (12)	5	4	2	1	42% 5/12	10**	7/8	0/1	2	6	0	0	4/8	51	9
III (5)	NA	NA	NA	NA	NA	8***	3/5	1/1	1	2	0	0	1/3	40	31
AL (34)	8	11	6	4	14/29		15/23	3/6	3	10	5	1	13/19	53	18

* 2 patients had only part 2 assessed.

** 11/12 patients were assessed.

*** probably false positive related to high score for quality of life (part 1) of two patients.

HL = hearing loss; NMDAS = Newcastle Mitochondrial Disease Adult Scale; MRS = magnetic resonance spectroscopy,

LA = lactic acidosis, MGCA = 3-methylglutaconic aciduria, MRI = magnetic resonance imaging; NA = not applicable.

GC-MS = Gas Chromatography–Mass Spectroscopy.

Degree of HL: mild HL<40 dB; moderate 41–70 dB; severe 71–90 dB; profound >91 dB.

Hearing deterioration was triggered by stressful situations (6 times), medications (aminoglycosides, 4 times), noise (2 times), or other major events such as acute pancreatitis or pregnancy (Table 3) with similar frequency in both the multi-symptomatic and isolated HL subgroups.

**Figure 2:**
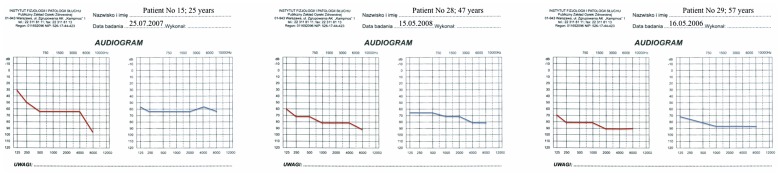
Subsequent audiograms of the patients presented in **Fig. 1**. Patient 15 has developed multisystem disorder; patients 28 and 29 have remained oligosymptomatic. Hearing loss has not progressed in patient 28.

**Figure 3:**
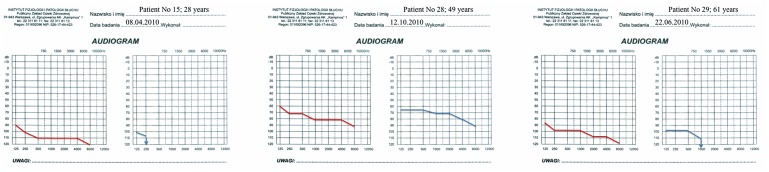
Latest audiograms of the three patients shown in **Figs. 1** and **2**. Patients 15 and 29 have become cochlear implant users. The HL threshold of patient 28 has still not changed.

The majority of audiograms showed a pantonal shape with high frequencies more severely affected (Figures 1, 2, and 3). BEAP did not show retro-cochlear pathology in any case and always confirmed audiometric hearing thresholds. Otoacoustic emissions were absent in all HL patients. Type A tympanograms and stapedial reflexes were registered in all ears (excluding severe and profound HL).

### Heteroplasmy study

**Figure 4:**
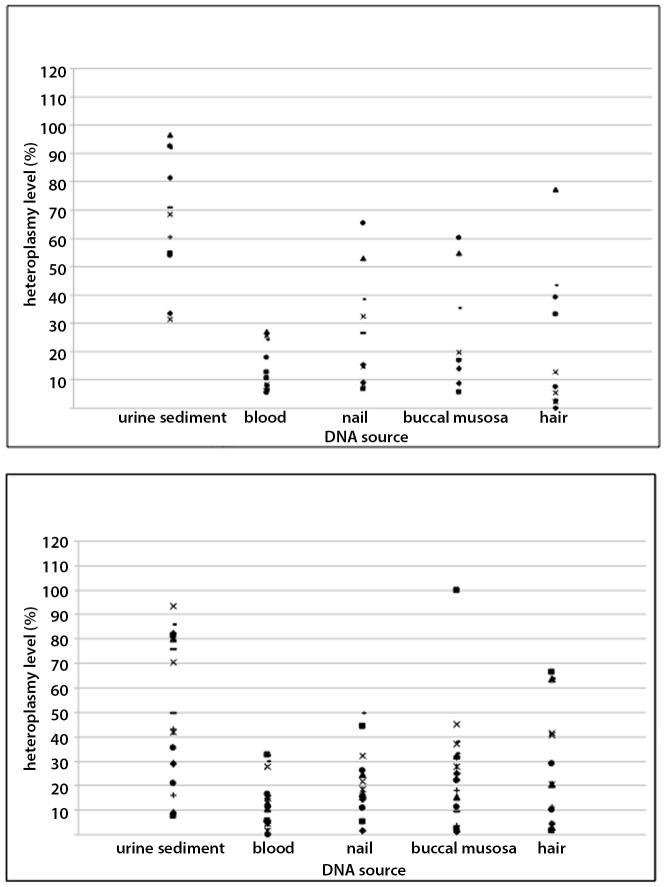
Heteroplasmy level of the m.3243A>G mutation in examined tissues. Above: the scores of patients with multi-symptomatic presentation (subgroup I). Below: the scores of patients with isolated hearing loss and asymptomatic carriers (subgroups II and III together).

The levels of heteroplasmy in the whole m.3243A>G group decreased in the following order: urine, hair, buccal mucosa, nails, and blood (Table 4). The mean level of heteroplasmy detected in urine (57.6%) was significantly higher (*p*<10^−6^) than in any other tested tissue, whereas blood heteroplasmy levels were significantly lower compared to other tissues (*p*<0.002, Table 4). Notably, the most pronounced mean difference (44.1%) was found in a comparison between urine and blood (*p*<10^−7^). Analysis of heteroplasmy levels among other tissues did not reveal significant differences (data not shown). Mean heteroplasmy load was relatively higher in the multi-systemic subgroup than in the isolated HL and asymptomatic subgroups (Table 4), but the differences were relatively small. The patients from the isolated HL and the asymptomatic subgroups showed low as well as high mtDNA load in all tissues studied (Figure 4).

After adjustment for age we detected a relatively weak correlation between total disease score and heteroplasmy level in urine (*p* = 0.034) or nails (*p* = 0.015), but the distribution of m.3243A>G mutation load among tissues and individuals did not allow clinically useful prediction of disease severity. There was no relation between kidney disease in the patients 9 and 16 (Table 3) and level of urine heteroplasmy [30].

### Brain imaging and lactates

Magnetic resonance imaging (MRI) was abnormal in 13 of 19 examined patients (Table 5). The most frequent changes were cerebellar atrophy and increased mineralisation of the globus pallidus. Chronic and/or acute stroke-like changes were seen in two cases. Cerebral abnormalities were found in MRI, mainly in the multi-symptomatic subgroup (in all examined patients), but also in the four patients from the isolated HL and one patient from the asymptomatic subgroups. Details of the MRI findings are given in Table 3.

Magnetic resonance spectroscopy (MRS) of the brain showed increased lactate signal in 6 out of 19 examined patients (Table 5). The pathology was seen only in the multi-symptomatic subgroup. The lactate signal was absent in three MRS scans, and was uncertain in 10 patients (Table 3). Profiling of urinary organic acids revealed an increased excretion of lactic acid in three patients and/or 3-methylglutaconic acid (3-MGCA) in six patients (Table 3).

## Discussion

In this hearing loss-oriented study, we found that about 1% of Polish patients presenting with isolated HL that had begun between the ages of 5 and 40 years harboured the m.3243A>G mutation in their blood DNA. This figure is lower than ∼2.4% reported for Japanese HI patients [20], [31], [32], but similar to that reported in a diabetic Caucasian cohort [33] and 3 times less than the prevalence of m.1555A>G mutation (3.4%) reported for HL patients [34]. A similar hearing loss study was carried out by Majamaa et al. who found m.3243A>G mutation in 2.1% of 250 HL patients by applying pre-selection based on maternal inheritance [16].

The association between isolated HL and m.3243A>G is consistent with the data of Manwaring et al. who studied hair follicle mtDNA in 900 elderly individuals and found six m.3243A>G carriers, all having mild to moderate hearing impairment [35]. Uimonen et al. showed that isolated HL associated with m.3243A>G mutation resembled presbyacusis, but its progression rate was faster [22].

The m.3243A>G HL in our study was not always progressive and remained the only complaint in 42% of patients (Table 3). Mitochondrial etiology of long-standing hearing loss in a 78 year-old woman was established after development of m.3243A>G associated MELAS phenotype in her son. Molecular testing for m.3243A>G mutation was positive in at least four young adults with stable HL which had lasted more than 10 years. Data on the non-progressive course of m.3243A>G induced long-lasting HL is limited. In the key paper of Uimonen et al., the progressive pattern was the most common, with high frequencies affected first [22]. A relationship between HL progression and severity of m.3243A>G phenotype has been found [23]. This suggests that a low progression rate of hearing impairment is a good prognostic marker.

The m.3243A>G positive subgroup differed from the whole study group by having relatively later onset of HL, as well as presenting maternal inheritance of HL in all except one case. However, at the first presentation of HL in m.3243A>G carriers, the risk of developing multisystem disease cannot be predicted.

Analysis of circumstances of hearing deterioration in m.3243A>G mutation revealed various triggering factors, some of which have been suggested previously, such as exposure to aminoglycosides, valproates, noise exposure, or stress [36], [37]. Other triggers, such as pregnancy or pancreatitis, have not been convincingly reported in the literature.

In this study the onset of m.3243A>G associated HL was earlier in men than in women (18 and 28 years, respectively). Uimonen et al. found that being male, as well as a high heteroplasmy level of the mutation, may aggravate the severity of hearing impairment in m.3243A>G carriers [22]. More epidemiological data are needed to properly assess a sex influence on the m.3243A>G phenotype.

In our patients the m.3243A>G mutation load was lowest in the DNA derived from archived blood samples compared to other analysed tissues. It has previously been found that the mtDNA mutation load in blood decreases in older patients due to the negative selection of leukocytes carrying impaired mitochondria [38], [39]; because of this, significantly better detection of m.3243A>G can be achieved by analysing urine [40], buccal mucosa, or hair [35], [36], [41], [42].

In this study, the heteroplasmy level of m.3243A>G mutation was markedly (2–4 times) higher in DNA derived from urine compared to other tissues. Interestingly, the urinary heteroplasmy of over 30% was found not only in the classical MELAS patients, but also in the oligosymptomatic and even asymptomatic m.3243A>G persons.

Our results are consistent with those of Whittaker et al. who showed that urine is the most suitable material for detection of the m.3243A>G mutation. The authors concluded that the screening of urine for m.3243A>G mutation load is a better predictor of outcome than the gold standard of skeletal muscle [40].

Short stature [43], abnormal excretion of 3-MGCA in urine [44], [45], and elevation of lactates in the brain MRS, which were seen in our oligo- and asymptomatic m.3243A>G carriers, may be helpful in identifying affected individuals, but these are not sensitive tests either.

### Summarizing

People with postlingual isolated HL require testing for m.3243A>G mutation. Early detection of patients with mitochondrial isolated HL and identification of mutation-carrying healthy relatives would improve proper prophylactic management – i.e. avoidance of harmful environmental factors (noise and stress) and certain medications (aminoglicosides and valproate). New therapies may become available in the near future. Urinary sediment – not blood – is the material of choice for assessing the mtDNA mutation load in people with isolated hearing loss. The possible prognostic value of high and low levels of mtDNA heteroplasmy in urine sediment needs further study.
